# Structure and work process in primary care and hospitalizations for sensitive conditions

**DOI:** 10.11606/S1518-8787.2017051007033

**Published:** 2017-08-03

**Authors:** Waleska Regina Machado Araujo, Rejane Christine de Sousa Queiroz, Thiago Augusto Hernandes Rocha, Núbia Cristina da Silva, Elaine Thumé, Elaine Tomasi, Luiz Augusto Facchini, Erika Barbara Abreu Fonseca Thomaz

**Affiliations:** I Programa de Pós-Graduação em Saúde Coletiva. Universidade Federal do Maranhão. São Luís, MA, Brasil; II Departamento de Saúde Pública. Universidade Federal do Maranhão. São Luís, MA, Brasil; III Centro de Pós-Graduação e Pesquisa em Administração. Universidade Federal de Minas Gerais. Belo Horizonte, MG, Brasil; IV Departamento de Medicina Social. Faculdade de Medicina da Universidade Federal de Pelotas. Pelotas, RS, Brasil

**Keywords:** Primary Health Care, organization & administration, Patient Admission, Outcome and Process Assessment (Health Care), Program Evaluation, Ecological Studies

## Abstract

**OBJECTIVE:**

The objective of this study is to investigate whether the characteristics of the structure of primary health units and the work process of primary care teams are associated with the number of hospitalizations for primary care sensitive conditions.

**METHODS:**

In this ecological study, we have analyzed data of Brazilian municipalities related to sociodemographic characteristics, coverage of care programs, structure of primary health units, and work process of primary care teams. We have obtained the data from the first cycle of the Brazilian Program for Improving Access and Quality of the Primary Care, of the Department of Information Technology of the Brazilian Unified Health System, the Brazilian Institute of Geography and Statistics, and the United Nations Development Programme. The associations have been estimated using negative binomial regression coefficients (β) and respective 95% confidence intervals, with a hierarchical approach in three levels (alpha = 5%).

**RESULTS:**

In the adjusted analysis for the outcome in 2013, in the distal level, the coverage of the *Bolsa Família* Program (β = -0.001) and private insurance (β = -0.01) had a negative association, and the human development index (β = 1.13), the proportion of older adults (β = 0.05) and children under the age of five (β = 0.05), and the coverage of the Community Health Agent Strategy (β = 0.002) showed positive association with hospitalizations for primary care sensitive conditions. In the intermediate level, minimum hours (β = -0.14) and availability of vaccines (β = -0.16) showed a negative association, and availability of medications showed a positive association (β = 0.16). In the proximal level, only the variable of matrix support (β = 0.10) showed a positive association. The variables in the adjusted analysis of the number of hospitalizations for primary care sensitive conditions in 2014 presented the same association as in 2013.

**CONCLUSIONS:**

The characteristics of the structure of primary health units and the work process of the primary care teams impact the number of hospitalizations for primary care sensitive conditions in Brazilian municipalities.

## RESUMO

**OBJETIVO:**

Investigar se características da estrutura das unidades básicas de saúde e do processo de trabalho das equipes de atenção básica estão associadas ao número de internações por condições sensíveis à atenção primária.

**MÉTODOS:**

Neste estudo ecológico, foram analisados dados de municípios brasileiros relativos a características sociodemográficas, de cobertura de programas assistenciais, de estrutura das unidades básicas de saúde e processo de trabalho das equipes de atenção básica. Os dados foram obtidos do primeiro ciclo do Programa de Melhoria do Acesso e Qualidade da Atenção Básica, do Departamento de Informática do Sistema Único de Saúde, do Instituto Brasileiro de Geografia e Estatística e do Programa das Nações Unidas. Estimaram-se as associações por meio de coeficientes de regressão binomial negativa (β) e respectivos intervalos de confiança a 95%, com abordagem hierarquizada em três blocos (alpha = 5%).

**RESULTADOS:**

Na análise ajustada, para o desfecho em 2013, no bloco distal, a cobertura do Programa Bolsa Família (β = -0,001) e de plano privado (β = -0,01) apresentaram associação negativa; e o índice de desenvolvimento humano (β = 1,13), a proporção de pessoa idosa (β = 0,05) e de menor de cinco anos (β = 0,05) e a cobertura da Estratégia de Agentes Comunitários de Saúde (β = 0,002) mostraram associação positiva com internações por condições sensíveis à atenção primária. No bloco intermediário, apresentaram associação negativa o horário mínimo (β = -0,14) e a disponibilidade de vacina (β = -0,16); e associação positiva, a disponibilidade de medicamentos (β = 0,16). No bloco proximal, apenas a variável apoio matricial (β = 0,10) mostrou associação positiva. Na análise ajustada do número de internações por condições sensíveis à atenção primária em 2014, as variáveis apresentaram o mesmo sentido de associação de 2013.

**CONCLUSÕES:**

Características da estrutura das unidades básicas de saúde e do processo de trabalho das equipes de atenção básica impactam no número de internações por condições sensíveis à atenção primária nos municípios brasileiros.

## INTRODUCTION

The rate of hospitalizations for primary care sensitive conditions (ICSAP) represents an indicator of access and quality of the primary health care (PHC)^17^ ^,^ ^21^ ^,^ ^23^. International studies have shown that factors associated with higher rates of ICSAP are: male gender, senility, living in rural areas, low income, and socioeconomic disadvantage^1^ ^,^ ^2^.

In Brazil, a cross-sectional study has identified a higher probability of ICSAP in females, children under five years of age, persons with less schooling, history of emergency medical consultation in the previous month, and hospitalization in the previous year^15^. In a cohort of adults, higher rates of ICSAP have been observed in persons over 60 years of age and non-white; however, no gender differences have been identified^8^.

There is evidence that the expansion of the Family Health Strategy (FHS) is associated with a tendency to reduce this indicator^4^ ^,^ ^7^ ^,^ ^10^ ^,^ ^12^. Therefore, although the rates of ICSAP are still high when compared to those of other countries^24^, there was a decrease of more than 5% per year between 2008 and 2014 in Brazil^12^. It is possible that only the increase in coverage of the FHS will not be sufficient for the consistent reduction of this indicator. However, few studies have analyzed the characteristics of the quality of the care involving elements of structure and work process in PHC.

The association of these characteristics with the indicators of ICSAP is not well established. Gonçalves et al.^8^ have found no association between quality of services in PHC and rates of ICSAP. A Spanish ecological study has found no significant association with the number of physicians per inhabitant, but it has shown that the physician workload has a significant direct association with sensitive hospitalizations, and it suggests that it is a result of the impairment of the quality of care offered to the patient^13^.

Therefore, given the need to better exam the relation between the characteristics of PHC and ICSAP, the hypothesis of this work is that the better the structure and the work process in PHC, the lower the number of ICSAP, even after adjusting for the sociodemographic variables and variables of coverage of care programs.

## METHODS

This study is ecological, with population data available on different databases. The study population consisted of 5,565 Brazilian municipalities, 38,812 Basic Health Units (BHU), and a sample of 17,202 primary care teams (PCT) existing in 2012. From these data, aggregate indicators were constructed for the municipal level.

All variables used in this study are described in the Figure. Details on the construction of the indicators are presented in the Chart. The outcome was the number of ICSAP for 2012 and 2013 obtained from the System of Hospitalizations (SIH) of the Brazilian Unified Health System (SUS). In addition, we calculated the rates and proportions of ICSAP from 2012 to 2014. The sociodemographic variables were obtained from IBGE^a^ and UNDP^b^: proportion of older population (> 60 years) and children under five years of age, Gini coefficient, and Municipal Human Development Index (HDI).

**Figure:**
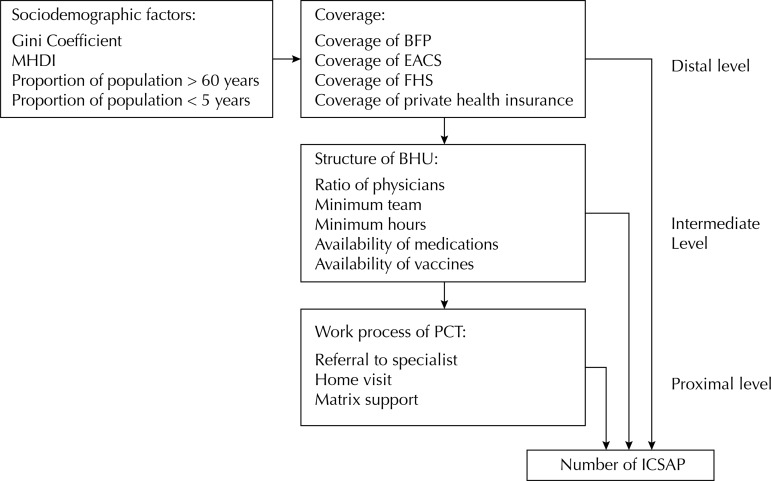
Scheme of the theoretical explanatory model for the investigation of factors associated with the rate of ICSAP in three hierarchical levels.

MHDI: Municipal Human Development Index; BFP *Bolsa Família* Program; EACS: Community Health Agent Strategy; FHS: Family Health Strategy; PCT: Primary Care Teams; BHU: Basic Health Units; ICSAP: Hospitalizations for primary care sensitive conditions

**Chart:** Description of the study indicators.

Indicator	Description	Source/Year
% of older adults in the population	Percentage of persons aged 60 years or over, in the total population living in a given geographical area, in the year considered.	Demographic Census (IBGE, 2010)
% of children under the age of five in the population	Percentage of persons aged 5 years or less, in the total population living in a given geographical area, in the year considered.	
Gini index of per capita household income	It measures the degree of concentration of the per capita household income distribution of a given population and in a given geographic space. The index varies from zero (0), perfect equality, to one (1), perfect inequality.	National Household Sample Survey – PNAD (IBGE, 2010)
Municipal Human Development Index (MHDI)	It is a measure composed of indicators of three dimensions of human development: longevity, education, and income. It varies from 0 to 1. The closer to 1, the greater the human development.	United Nations Development Programme – UNDP (2010)
Rate of coverage of private health insurance	Ratio between the number of beneficiaries of private health insurance and the population of the area, multiplied by 100.	National Health Agency (2010)
% of BHU in the municipality with ratio of physicians/team ≥ 1	The number of physicians was obtained by adding the physicians of the minimum and extended team of each BHU of the municipality, divided by the total number of teams in each unit. The indicator was then categorized to reflect the average percentage of BHU of the municipality with at least one physician per team.	Module I of cycle 1 of the PMAQ-AB (2012)
% of BHU in the municipality with minimal health team	It is recommended that the minimum team should be composed of physician, nurse, nursing assistant and technician, and four Community Health Agents (CHA)^5^. The minimum team indicator corresponded to the average percentage of BHU of the municipality with minimum teams.	
% of BHU in the municipality operating at least the minimum hours	Hours of operation were considered minimum when the BHU operated at least two shifts a day, five days a week. An indicator was constructed for the municipality calculating the average percentage of BHU with minimum hours.	
% of BHU in the municipality with availability of at least 75% of the 12 vaccines of the basic calendar	The availability of vaccines was estimated by the existence and availability of immunobiological agents advocated by the National Immunization Policy. The indicator corresponded to the average percentage of BHU that always had more than 75% of the 12 vaccines of the basic calendar. The cutoff point refers to the proportion of adequate coverage^a^.	
% of BHU in the municipality with 80% or more of the medications of the basic component of the National Essential Medicine List (RENAME)	The medications of the basic component of the RENAME were verified, in sufficient quantity (antiparasitics, multivitamins and minerals, antiasthmatics, hormones, antihypertensives and of cardiovascular action, antidiabetics, antibacterials, analgesics/antipyretics, treatment and prevention of osteoporosis, antacids/antiemetics/antisecretors). The indicator refers to the sum of medications available in sufficient quantity, divided by the total number of medications in the list, multiplied by 100. The cutoff point refers to the goal of 80% availability of medications^b^.	
% of PCT in the municipality responsible for the user’s referral to specialized care	Percentage of PCT that, when a user needs to be referred to a specialist, schedules and informs the date of appointment with the specialist to the user.	Module II of cycle 1 of the PMAQ-AB (2012)
% of PCT in the municipality with home visit	The indicator of home visit for the municipality represents the average percentage of teams that reported visits, taking into account the CHA and other team members.	
% of PCT in the municipality with matrix support	The matrix support is a methodology of care management and is a technical and pedagogical support to teams^6^. This indicator represented the average percentage of teams that reported receiving support from other professionals to solve complex cases.	
Rate of ICSAP^c^	Ratio between the number of ICSAP and the population of the municipality per 10,000 inhabitants.	SIH-SUS (2012 to 2014), Demographic Census (IBGE, 2010)
Proportion of ICSAP^c^	Percentage of ICSAP in relation to total hospitalizations in the municipality for each year.	
Number of ICSAP^c^	Absolute number of ICSAP.	

%: Percentage frequency; IBGE: Brazilian Institute of Geography and Statistics; IDHM: Municipal Human Development Index; RENAME: National Essential Medicine List; PMAQ-AB: National Program for Improving Access and Quality of the Primary Care; BHU: Basic Health Units; PCT: Primary Care Teams; CHA: Community Health Agents; ICSAP: Hospitalizations for primary care sensitive conditions; SIH-SUS: Hospital Information System of the Brazilian Unified Health System

^a^ Ministério da Saúde. Secretaria de Gestão Estratégica e Participativa. Departamento de Articulação Interfederativa. Caderno de Diretrizes, Objetivos,

Metas e Indicadores: 2013-2015. Brasília (DF); 2013 [cited 2015 Sep 18]. Available from: http://bvsms.saude.gov.br/bvs/publicacoes/caderno_diretrizes_objetivos_2013_2015.pdf

^b^ World Health Organization. Global action plan for the prevention and control of noncommunicable diseases 2013-2020. WHO: Geneva; 2013 [cited 2015 Sep 18]. Available from: http://www.who.int/global-coordination-mechanism/publications/global-action-plan-ncds-eng.pdf

^c^ Hospitalizations were selected by place of residence, according to ICD-10, in the Hospital Information System. The following hospitalizations were excluded: long stay, pregnancy, delivery, and postnatal. The diagnoses of hospitalizations were selected based on the Brazilian List of ICSAP^3^. Brazilian List of Hospitalizations for Primary Care Sensitive Conditions published by SAS/MS # 221, of April 17, 2008.

The coverage indicators of municipal care programs – coverage of the *Bolsa Família* Program (BFP), Community Health Agent Strategy (EACS), and FHS – were obtained from the Primary Care Information System (SIAB).

The structure data of the BHU came from module I of the first cycle of the External Evaluation of the National Program for Improving Access and Quality of the Primary Care (PMAQ-AB), from on-site observation. The work process in PHC was obtained by interviews with the PCT that were part of the PMAQ-AB. The work process variables were referral to specialist, home visit, and matrix support. The PMAQ-AB data were collected by electronic questionnaire using tablets^c^. After data collection, the data were validated and sent to the database centralized by the Ministry of Health, which later made them available on the website of the Department of Primary Care^c^.

For the statistical analysis of the data, we used the software Stata 12.0 (Stata Corp., College Station, TX, USA). Initially, three databases were tabulated. The first one corresponded to the data of the municipalities, the second one, to the questions of Module I of the PMAQ-AB, and the third one, to the work process variables of the PCT. Then, the structure and work process variables were aggregated for the municipal level, estimating the average percentage of variables per municipality. The variable used to link the data was the IBGE code.

The descriptive analysis estimated: averages (X) ± standard deviations (SD), as well as medians (Md) and interquartile deviations (Q1 and Q3), in addition to minimum (min) and maximum (max) values.

Univariate and multivariate analyses were performed using negative binomial regression considering municipal population. The regression coefficients (β) and respective 95% confidence intervals (IC95%) were calculated.

For the modeling, we used a hierarchical approach in three levels. The distal level corresponded to the sociodemographic variables and variables of coverage of care programs. The intermediate level presented the structural variables of the BHU in the municipalities. The proximal level consisted of the characteristics of the work process of PCT (Figure).

In the adjusted analysis, all variables of the distal level were initially included in the model, remaining the variables with p < 0.10. Then, the variables of the intermediate level were included in the model, and we removed the variables of this level with p > 0.10, adjusted for the variables that came from the previous level. The same procedure was repeated for the proximal level, adjusted for the variables of the previous levels.

Alpha of 5% was used as a criterion of statistical significance. To this end, the regression estimates and 95%CI were verified in their corresponding level. All analyses considering the variables of the proximal level (work process) were weighted for unequal selection probabilities, as we used the entire population in the previous levels (municipalities and BHU), but only one sample in the proximal level (by auto-inclusion). As the proportion of adherence of the PCT to PMAQ-AB was different among the Brazilian municipalities, the estimates were weighted by the inverse of the probability of adherence. This variable was constructed as the ratio of total existing PCT by the number of teams that were part of the program in the municipality.

In compliance with the Resolution of the National Health Council, the study was approved by the Ethics Committee of the Universidade Federal de Pelotas (Official Letter 38/12, in May 10, 2012). All persons interviewed signed the informed consent.

## RESULTS

Among the indicators of the distal level, the average Gini coefficient of 0.5 and average HMDI of 0.6 (both with SD = 0.07) can be highlighted. The proportion of older population had an average of 12.1% and children under the age of five had an average of 7.4%. The coverage of the BFP presented an average of 80.7% (SD = 18.1%), the EACS, an average of 90.2% (SD = 23.2%), and the FHS, an average of 81.9% (SD = 29%). The average rate of private health insurance in the municipalities was 8.4% (SD = 11.4%) (Table 1).

**Table 1:** Descriptive characteristics of the independent variables at the three levels of analysis, in 2012. PMAQ-AB, cycle 1.

Variable	x̄	SD	Q1	Med.	Q3	Min.	Max.
Distal level (municipality)							
Gini Coefficient	0.5	0.07	0.4	0.5	0.5	0.3	0.8
MHDI	0.6	0.07	0.6	0.7	0.7	0.4	0.9
% population ≥ 60 years	12.1	3.3	9.9	12.1	14.2	2.6	29.4
% population < 5 years	7.4	1.8	6.2	7.2	8.3	2.3	19.4
Coverage of BFP	80.7	18.1	72.2	85.1	94.7	0	100
Coverage of EACS	90.2	23.2	100	100	100	0	100
Coverage of FHS	81.9	29.0	71.6	100	100	0	100
Coverage of private insurance	8.4	11.4	1.1	3.6	10.9	0.01	96.11
Intermediate level (BHU)							
BHU ≥ 1 physician/team^a^	81.8	26.9	69.8	100.0	100	0	100
Minimum team^b^	65.0	32.0	45.0	66.7	100	0	100
Minimum hours^c^	87.1	22.3	80.0	100	100	0	100
Availability of medications >80%^d^	4.1	16.6	0	0	0	0	100
Availability of vaccines >75%^e^	57.1	35.5	28.6	57.1	100	0	100
Proximal level (PCT)							
Referral to specialist^f^	56.6	43.0	0	66.7	100	0	100
With home visit^g^	99.3	6.9	100	100	100	0	100
With matrix support^h^	84.1	30.9	84.3	100	100	0	100

PMAQ-AB: National Program for Improving Access and Quality of the Primary Care; MHDI: Municipal Human Development Index; BFP: *Bolsa Família* Program; EACS: Community Health Agent Strategy; FHS: Family Health Strategy; PCT: primary care team; BHU: basic health unit; x̄: mean; Q1: 1st quartile; Med.: median; Q3: 3rd quartile; Min.: minimum; Max.: maximum; %: percentage frequency; RENAME: National Essential Medicine List

^a^ Average number of municipalities with BHU with at least 1 physician in the team.

^b^ Average number of municipalities with at least the minimum team (1 physician, 1 nurse, 1 nursing technician or assistant, and 4 community health agents) at the BHU.

^c^ Average number of municipalities with BHU with at least two shifts for five days/week.

^d^ Average number of municipalities with BHU with at least 80% of RENAME medication in sufficient quantity.

^e^ Average number of municipalities with BHU with at least 75% of the vaccines of the basic calendar.

^f^ Average number of municipalities with teams that guarantee the referral of the user to a specialist.

^g^ Average number of municipalities with teams that carry out home visit.

^h^ Average number of municipalities with teams that receive matrix support.

In 2012, Brazilian municipalities had, on average, at least one physician/team in 81.8% of the BHU. The average percentage, per municipality, of BHU with complete minimum team was 65.0%, those that operated at minimum hours was 87.1%, and those with at least 75% of immunobiological agents was 57.1%. The worst indicator of this level was the availability of medications: only an average of 4.1% of BHU per municipality had 80% or more of the medications available. Of the PCT that were part of the PMAQ-AB, on average, 56.6% of the teams per municipality guaranteed referral to specialist, 99.3% carried out home visits, and 84.1% received matrix support (Table 1).

The absolute number, rate, and proportion of ICSAP decreased, as shown in Table 2. The average rate of ICSAP went from 169.2 (SD = 128.5) in 2012 to 154.3/10,000 inhabitants (SD = 122.1) in 2014 (Table 2).

**Table 2:** Descriptive analysis of hospitalizations for primary care sensitive conditions in 2012, 2013, and 2014.

ICSAP	x̄	SD	Q1	Med.	Q3	Min.	Max.
Number of ICSAP (2012)	433.8	1643.1	57.0	155.5	434.0	1.0	88291.0
Number of ICSAP (2013)	428.0	1636.4	57.0	155.0	442.0	1.0	90379.0
Number of ICSAP (2014)	407.3	1582.8	53.0	142.0	414.0	2.0	88212.0
Rate of ICSAP/10,000 (2012)	169.2	128.5	77.5	132.9	224.9	0.9	997.9
Rate of ICSAP/10,000 (2013)	161.3	121.8	74.7	126.2	212.6	1.7	990.3
Rate of ICSAP/10,000 (2014)	154.3	122.1	69.1	116.9	202.2	1.4	960.9
Proportion of ICSAP (2012)	30.5	12.7	20.9	28.4	38.2	2,2	86.1
Proportion of ICSAP (2013)	29.9	12.5	20.6	27.7	37.3	1.7	84.8
Proportion of ICSAP (2014)	28.1	12.3	19.1	25.7	35.2	3.7	85.0

ICSAP: Hospitalization for primary care sensitive conditions; x̄: mean; Q1: 1st quartile; Med.: median; Q3: 3rd quartile; Min.: minimum; Max.: maximum

The unadjusted analysis for outcome in 2013 and 2014 showed a similar association. There was a significant negative association between the proportion of children under five years of age, coverage of private health insurance, minimum hours, and availability of vaccines. There was a significant positive association between the proportion of persons aged 60 years or over, coverage of BFP, EACS, and FHS, availability of medications, and matrix support (Table 3).

**Table 3:** Unadjusted and adjusted analysis of the factors associated with the number of ICSAP in 2013 and 2014, in the distal, intermediate, and proximal levels.

Variable	Number of ICSAP (2013)						Number of ICSAP (2014)					
	
	Unadjusted			Adjusted			Unadjusted			Adjusted		
			
	Coeff^a^	95%CI^a^	p^a^	Coeff^a^	95%CI^a^	p	Coeff^a^	95%CI^a^	P^a^	Coeff^a^	95%CI^a^	p^a^
Distal level												
Gini Coefficient	-0.10	-0.40–0.19	0.485				-0.27	-0.40–0.19	0.070.			
MHDI	-0.06	-0.33–0.21	0.673.	**1.13**	**0.69–1.57**	**< 0.001**	0.08	-0.19–0.36	0.565	**1.30**	**0.85–1.75**	**< 0.001**
% > 60 years	0.04	0.03–0.04	< 0.001	**0.05**	**0.04–0.06**	**< 0.001**	0.04	0.03–0.04	< 0.001	**0.05**	**0.04–0.06**	**< 0.001**
% < 5 years	-0.03	-0.04– -0.02	< 0.001	**0.05**	**0.03–0.07**	**< 0.001**	-0.03	-0.04– -0.02	< 0.001	**0.05**	**0.03–0.07**	**< 0.001**
Coverage BFP	0.001	0.0001–0.002	0.029	**-0.001**	**-0.002– -0.0001**	**0.033**	0.001	0.0001–0.002	0.035	**-0.001**	**-0.002– -0.0002**	**0.017**
Coverage EACS	0.003	0.002–0.004	< 0.001	**0.002**	**0.001–0.003**	**< 0.001**	0.003	0.002–0.004	< 0.001	**0.002**	**0.001–0.003**	**< 0.001**
Coverage FHS	0.002	0.001–0.003	< 0.001				0.002	0.001–0.003	< 0.001			
Coverage private insurance	-0.01	-0.01– -0.007	< 0.001	**-0.01**	**-0.01– -0.01**	**< 0.001**	-0.009	-0.01– -0.008	< 0.001	**-0.01**	**-0.01– -0.008**	**< 0.001**
Intermediate level												
% BHU ≥ 1 physician/team^b^	-0.02	-0.09–0.05	0.520				-0.02	-0.09–0.05	0.535			
% BHU minimum team^c^	0.04	-0.02–0.1	0.187				0.02	-0.04–0.08	0.461			
% BHU minimum hours^d^	-0.18	-0.26– -0.1	< 0.001	**-0.14**	**-0.23– -0.06**	**0.001**	-0.12	-0.21– -0.03	0.006	**-0.09**	**-0.18– -0.01**	**0.035**
% BHU ≥ 80% medications^e^	0.14	0.03–0.26	0.016	**0.16**	**0.04–0.27**	**0.007**	0.16	0.04–0.28	0.006	**0.17**	**0.05–0.28**	**0.004**
% BHU ≥ 75% vaccines^e^	-0.14	-0.20– -0.09	< 0.001	**-0.16**	**-0.22– -0.11**	**< 0.001**	-0.16	-0.21– -0.10	< 0.001	**-0.18**	**-0.24– -0.13**	**< 0.001**
Proximal level												
% referral by FHS^g^	-0.01	-0.07–0,05	0.684				-0.01	-0.07–0,05	0.827			
% PCT home visit^h^	0.19	-0.14–0.53	0.256				0.05	-0.33–0.44	0.777			
% PCT matrix support^i^	0.11	0.03–0.20	0.009	**0.10**	**0.02–0.19**	**0.015**	0.09	0.004–0.18	0.041	0.07	-0.01–0.16	0.092

ICSAP: Hospitalization for Primary Care Sensitive Conditions; MHDI: Municipal Human Development Index; BFP *Bolsa Família* Program; EACS: Community Health Agent Strategy; FHS: Family Health Strategy; PCT: Primary Care Team; BHU: Basic Health Units; x̄: mean; Q1: 1st quartile; Med.: median; Q3: 3rd quartile; Min.: minimum; Max.: maximum; %: percentage frequency; RENAME: National Essential Medicine List

^a^ Estimates calculated for the municipality level.

^b^ Average number of municipalities with BHU with at least 1 physician in the team.

^c^ Average number of municipalities with at least the minimum team (1 physician, 1 nurse, 1 nursing technician or assistant, and 4 community health agents) at the BHU.

^d^ Average number of municipalities with BHU with at least two shifts for five days/week.

^e^ Average number of municipalities with BHU with at least 80% of RENAME medication in sufficient quantity.

^f^ Average number of municipalities with BHU with at least 75% of the vaccines of the basic calendar.

^g^ Average number of municipalities with teams that guarantee the referral of the user to a specialist.

^h^ Average number of municipalities with teams that carry out home visit.

^i^ Average number of municipalities with teams that receive matrix support.

Values with statistical significance are presented in bold.

In the adjusted analysis of 2013, in the distal level, municipalities with higher coverage of BFP (β = -0.001) and higher coverage of private insurance (β = -0.01) had a lower number of ICSAP. Municipalities with higher MHDI (β = 1.13), higher proportion of older persons (β = 0.05), higher proportion of children under the age of five years (β = 0.05), and higher coverage of EACS (β = 0.002) showed a higher number of hospitalizations.

In the intermediate level, municipalities with BHU operating at least with minimum hours (β = -0.14) and with at least 75% of available vaccines (β = -0.16) presented a lower number of ICSAP. Municipalities with at least 80% of available medications showed a higher number of hospitalizations (β = 0.16).

In the proximal level, only the variable of matrix support (β = 0.10) showed a positive and significant association, which indicates that municipalities with more teams that receive matrix support have a higher number of hospitalizations (Table 3).

In the adjusted analysis of 2014, the variables of the distal and intermediate level presented the same association as in the adjusted analysis of 2013. The matrix support lost statistical significance only in the proximal level (β = 0.10, p = 0.092) (Table 3).

## DISCUSSION

After adjusting for the sociodemographic variables and variables of coverage of care programs, the variables of structure of the BHU (minimum hours, and availability of medications and vaccines) and the work process of the PCT (matrix support) were associated with the number of ICSAP.

The indicators of ICSAP decreased as it has been found in other studies, which have related this reduction to a better coverage of health services in recent years^7^ ^,^ ^11^ ^,^ ^17^. A large dispersion of this indicator was observed in the municipalities. The number of ICSAP presents a significant geographic variation^3^, what makes our study appropriate because of the uncovering of the characteristics of the primary health services associated with the number of ICSAP.

Regarding the care programs analyzed, although the coverage of FHS was not associated with the lowest indicators of ICSAP and the coverage of EACS showed an association with a higher number of hospitalizations, other articles show that the implementation of FHS seems to be associated with reduced ICSAP^11^ ^,^ ^12^. Dourado et al.^7^ show that socioeconomic variables reduce the impact of FHS in reducing hospitalizations.

These strategies are the main sources of access to primary services^d^; therefore, we expected to find a lower number of ICSAP in municipalities with higher coverage. Two possible explanations are suggested: first, the coverage of the FHS in the country may be overestimated, and second, the result found may reflect the higher frequency of health problems in the population of municipalities covered by the EACS, since it is aimed at more vulnerable locations with high rates of exclusion in the access to health services and within a disarticulated care network with poor structure^24^, which would justify more hospitalizations. For the moment, it is important to recognize the articulating role of community health agents in primary health care and reinforce the continuing education and supervision activities of their actions to ensure the effectiveness of their practices^5^.

The significant inverse association with BFP suggests that municipalities with a higher percentage of families that are part of income transfer programs have fewer hospitalizations. The descriptive analysis shows that, on average, 80% of families of municipalities with criteria for the monitoring of the BFP are covered by the program, ensuring a better income condition for the poor and extremely poor families benefited. Given the characteristics of the BFP, we highlight that its coverage is an important indicator of social vulnerability, with the population exposed to a diversity of situations of poverty and social inequalities^24^. This population may reside in municipalities with a specialized health services network of poor structure, so that services are not accessed because of a lack of supply, and not because of a lack of need of individuals^24^. Thus, a study suggests that the resolution of socioeconomic problems in poorer areas can impact the number of ICSAP^2^.

As the hospitalizations computed refer only to public hospitalizations, we already expected that municipalities with higher average coverage of private health insurance would have fewer hospitalizations as they are diverted to the private network. This result has been verified. The higher number of hospitalizations in municipalities with a higher proportion of older adults and children under five years of age was also found in another study, which shows higher rates of ICSAP at these extreme ages^15^.

Regarding the indicators of structure of the BHU, we did not identify an association between the ratio of physicians and ICSAP. The association with this indicator is inconclusive in the literature and should be analyzed with care, as it does not reflect the potential number of users per physician^16^. When assessing structural aspects, a systematic review has found an association between longer physician workloads and higher rates of ICSAP^23^. The authors have also identified that the availability of nurses decreases the rate of hospitalization for some diagnoses, such as asthma, and increases the rate for diabetes, although this risk decreases when care is accompanied by a community health agent^23^.

There was an association between the minimum hours of operation of the BHU and lower number of hospitalizations. A systematic review shows that most studies find an inverse association between access indicators and ICSAP^15^ ^,^ ^21^.

Among the hospitalizations that can be avoided, we can mention those resulting from vaccine-preventable diseases. The increase in vaccine coverage has reduced the infant morbidity and mortality of target diseases^9^. Therefore, we can explain the lower number of ICSAP in municipalities with at least 75% of immunobiological agents available, because of the better availability of vaccines.

The availability of medications was the worst indicator of structure. The unavailability of medications impairs the access to this resource and supply is still insufficient and unequal in Brazil, despite the National Essential Medicine List (RENAME) having increased free access^18^. The fragility in the selection and use of RENAME in many Brazilian municipalities can justify this finding and alert managers to invest in pharmaceutical care management^14^.

The adjusted regression analysis showed that the number of ICSAP was higher in municipalities with at least 80% of medications available, although access to them and their rational use are essential to avoid hospitalizations and deaths^e^. This study shows an increase in the number of hospitalizations from drug side effects and intoxications and shows an increasing tendency of problems related to the use of medications by older adults^21^, but this evidence is not enough to explain the higher number of hospitalizations. We suggest that the indicator of availability of medications is not the most adequate to explain the number of ICSAP, because it does not measure access or use of medications, besides the possibility of reverse causality – first there is the hospitalization and then the need for medication use. In addition, better structured municipalities would have a greater supply of health services and supplies, including medical appointments, medications, and hospital beds. The association observed may result from the possibility of access to hospital beds in municipalities with better structure of health services. It should also be noted that the FHS has great difficulty in asserting itself as a priority model, because of the high complexity supply and hospital coverage offered in these municipalities^24^.

From the characteristics of the work process, the matrix support that the FHS team received was the only one that showed direct association with outcome. This management tool provides specialized support to PCT, but the scarcity of specialized services induces its misuse in a substitute way, which can distort its function^6^.

An important activity of the care team is to guarantee to the users the care of other care points when necessary^22^. As almost all teams (99.6%) reported home visits, the indicator may not have been able to identify associations. However, this result, coupled with the non-association of ICSAP with the referral of the user to a specialist and the opposite association with matrix support, leads us to question the quality of the work process of the teams.

A systematic review^23^ provides strong evidence that the continuity of the care decreases the risk of ICSAP at all ages and in different countries. However, it points out the lack of evidence of the positive effect of other organizational aspects of the PHC in ICSAP^23^.

A cohort study in Southern Brazil has concluded that primary services with less than ideal quality appear to have no impact on ICSAP. The authors suggest that, in order to reduce ICSAP, investments need to be made on the quality of the PHC and the reorganization of the work process, effectively turning the PHC into the coordinator of the network^8^.

This study had some limitations. The regression analysis was not stratified by age group for ICSAP or by type of hospitalization. However, it was adjusted for the proportion of extreme ages. However, we suggest future stratified analyses to better understand the relations in question.

Data from the Health Information Systems have weaknesses both in relation to the hospitalization diagnosis and the coding of diagnosis using the ICD. However, because they present a good proportion of valid information, they are considered as important databases^20^.

The data were collected by previously trained evaluators with validation and checking strategies. In spite of this, the collection was made from the observation of BHU in module I and it may present problems of standardization. The data of module II were collected from interviews with a professional of the PCT, a situation that can lead to bias as it was an evaluation process.

Questions related to work process may be overestimated, since adherence to the PMAQ-AB may be conditioned by a better situation of the health teams. In an attempt to balance the difference in adherence among municipalities, we weighted the variables of the proximal level using the variable weight in the regression analysis. For the municipal and BHU variables, there was no selection bias, since data were collected for the entire study population.

The study had a large sample, which ensures greater precision of the estimates. Negative binomial regression allows the adjustment of the number of hospitalizations by the size of the population of the municipality, and the hierarchical regression analysis in three levels allowed the reduction of confounding bias.

An ecological study does not allow inferences at the individual level. The interest of the ecological effect in this study is to verify if ICSAP result from interactions between the sociodemographic factors and the factors of coverage of care programs, structure, and work process of the PHC.

There are apparently contradictory results, such as the association of the lower number of ICSAP with the indicators of “municipalities with at least 80% of available medications” and “municipalities with higher population with BFP”. However, the indicators indicate municipalities with infrastructure problems in the supply of services and materials (medications). If municipalities do not offer these resources and have few hospital beds, consequently, there will be fewer hospitalizations, because persons will have more barriers to overcome and to obtain access to these resources (medications and hospitalizations) in places farther away from where they live. Thus, the findings are plausible.

We conclude that the characteristics of the structure of BHU and the work process of PCT impact the number of ICSAP in Brazilian municipalities. Investments aimed at the qualification of actions in PHC will potentially reduce ICSAP.

## Footnotes

a Ministério da Saúde (BR), DATASUS. Informações de Saúde - TABNET: demográficas e socioeconômicas. Dados oriundos do Censo Demográfico de 2010 e projeções intercensitárias do IBGE. [cited 2015 Sep 18]. Available from: http://www2.datasus.gov.br/DATASUS/index.php?area=0206

b Programa das Nações Unidas para o Desenvolvimento; Instituto de Pesquisa Econômica Aplicada; Fundação João Pinheiro. Índice de Desenvolvimento Humano Municipal Brasileiro. Brasília (DF): PNUD, IPEA, FJP, 2013 [cited 2015 Sep 18]. (Série Atlas do Desenvolvimento Humano no Brasil 2013). Available from: http://www.br.undp.org/content/brazil/pt/home/library/idh1/o-idh-brasileiro0.html

c Ministério da Saúde(BR), Secretaria de Asssitência à Saúde, Departamento de Atenção Básica. Programa Nacional de Melhoria do Acesso e da Qualidade da Atenção Básica (PMAQ): manual instrutivo*.* Brasília (DF); 2012 [cited 2015 Sep 18]. Available from: http://dab.saude.gov.br/portaldab/biblioteca.php?conteudo=publicacoes/pmaq

d Ministério da Saúde (BR). Portaria Nº 2.488 de 21 de outubro de 2011. Aprova a Política Nacional de Atenção Básica, estabelecendo a revisão de diretrizes e normas para a organização da Atenção Básica, para a Estratégia Saúde da Família (ESF) e o Programa de Agentes Comunitários de Saúde (PACS), Brasília (DF); 2011 [cited 2015 Sep 18]. Available from: http://bvsms.saude.gov.br/bvs/saudelegis/gm/2011/prt2488_21_10_2011.html

e World Health Organization. Promoting rational use of medicines: core components. Geneva: WHO; 2002 [cited 2015 Sep 18]. (WHO Police Perspectives on Medicines, 5). Available from: http://apps.who.int/iris/bitstream/10665/67438/1/WHO_EDM_2002.3.pdf
